# High-quality-draft genome sequence of the fermenting bacterium *Anaerobium acetethylicum* type strain GluBS11^T^ (DSM 29698)

**DOI:** 10.1186/s40793-017-0236-4

**Published:** 2017-02-20

**Authors:** Yogita Patil, Nicolai Müller, Bernhard Schink, William B. Whitman, Marcel Huntemann, Alicia Clum, Manoj Pillay, Krishnaveni Palaniappan, Neha Varghese, Natalia Mikhailova, Dimitrios Stamatis, T. B. K. Reddy, Chris Daum, Nicole Shapiro, Natalia Ivanova, Nikos Kyrpides, Tanja Woyke, Madan Junghare

**Affiliations:** 1 Department of Biology, Microbial Ecology, University of Konstanz, D-78457 Konstanz, Germany; 2 Konstanz Research School of Chemical Biology, University of Konstanz, D-78457 Konstanz, Germany; 3 Department of Microbiology, University of Georgia, Athens, GA USA; 4 DOE-Joint Genome Institute, Walnut Creek, CA USA

**Keywords:** Anaerobic, Gluconate, Glycerol, Microcompartments, *Lachnospiraceae*, *Firmicutes*, Gram-staining positive, Embden-Meyerhoff-Parnas pathway, Entner-Doudoroff pathway, Ferredoxin, Transporters

## Abstract

*Anaerobium acetethylicum* strain GluBS11^T^ belongs to the family *Lachnospiraceae* within the order *Clostridiales*. It is a Gram-positive, non-motile and strictly anaerobic bacterium isolated from biogas slurry that was originally enriched with gluconate as carbon source (Patil, et al., *Int J Syst Evol Microbiol* 65:3289-3296, 2015). Here we describe the draft genome sequence of strain GluBS11^T^ and provide a detailed insight into its physiological and metabolic features. The draft genome sequence generated 4,609,043 bp, distributed among 105 scaffolds assembled using the SPAdes genome assembler method. It comprises in total 4,132 genes, of which 4,008 were predicted to be protein coding genes, 124 RNA genes and 867 pseudogenes. The G + C content was 43.51 mol %. The annotated genome of strain GluBS11^T^ contains putative genes coding for the pentose phosphate pathway, the Embden-Meyerhoff-Parnas pathway, the Entner-Doudoroff pathway and the tricarboxylic acid cycle. The genome revealed the presence of most of the necessary genes required for the fermentation of glucose and gluconate to acetate, ethanol, and hydrogen gas. However, a candidate gene for production of formate was not identified.

**Electronic supplementary material:**

The online version of this article (doi:10.1186/s40793-017-0236-4) contains supplementary material, which is available to authorized users.

## Introduction

Strain GluBS11^T^ (= DSM 29698) is the type strain of the newly described species *Anaerobium acetethylicum* [1]. The genus *Anaerobium* belongs to the family *Lachnospiraceae* [2] within the class *Clostridia* [3] of the order *Clostridiales* [4] that is largely synonymous with *Clostridium* rRNA cluster XIVa [5, 6]. Members of the family *Lachnospiraceae* have been isolated from diverse habitats, but are mainly constituents of mammalian intestinal microbiota, especially from ruminants [7] and humans [8]. They are strictly anaerobic and primarily non-spore forming [9], and ferment polysaccharides to short-chain fatty acids such as acetate and propionate as fermentation products [10], e.g., *Eubacterium rectale* ATCC 33656 ^T^, *Eubacterium ventriosum* ATCC 27560 ^T^, *Coprococcus* sp. and *Roseburia* sp. [11, 12]. The family *Lachnospiraceae* as currently described in the National Center for Biotechnology Information homepage comprises 41 named genera and several unclassified isolates, of which a total of 143 draft or complete genome sequences are available. Strain GluBS11^T^ was isolated due to its ability to ferment gluconate, and the species epithet ‘*acetethylicum*’ refers to its main fermentation products acetate and ethanol during gluconate fermentation [1]. Within the diverse family of *Lachnospiraceae*, strain GluBS11^T^ is phylogenetically closely related to the type strains of *C. herbivorans* strain 54408 [94% 16S rRNA sequence similarity); [13], *C. populeti* ATCC 35295 ^T^ (93.3% similarity); [14], *Eubacterium uniforme* ATCC 35992 ^T^ (92.4% similarity), and *C. polysaccharolyticum* ATCC 33142 ^T^ (91.5% similarity); [15, 16]. Of these, all strains were reported to ferment sugars mainly to butyrate plus formate, acetate, ethanol or lactate, except *E. uniforme* *,* which does not produce butyrate. Similar to *E. uniforme* ATCC 35992 ^T^, strain GluBS11^T^ does not produce butyrate during the fermentation of sugars or glycerol [1, 17]. Moreover, none of the above strains except for strain GluBS11^T^ was tested for fermentation of gluconate.

The most prominent feature of *A. acetethylicum* strain GluBS11^T^ is its ability to ferment sugars (including oxidized sugar such as gluconate) and glycerol mainly to acetate, ethanol, hydrogen, and formate [1, 17]. Therefore, we selected strain GluBS11^T^ as a candidate for studying its potential to ferment gluconate or glycerol. Moreover, most of the described bacterial glycerol fermentations lead to 1,3-propanediol [18] and other undesired products such as butyrate or 2,3-butanediol. In contrast to this, strain GluBS11^T^ ferments glycerol mainly to ethanol and hydrogen gas as well as negligible amounts of acetate and formate [17]. Here we present the summary of the taxonomic classification and the features of *A. acetethylicum* strain GluBS11^T^ together with the description of the genome sequencing and annotation. Emphasis is given on understanding the central metabolism and fermentation pathways. The putative enzymes involved in the fermentation of gluconate, glucose, and glycerol were also reconstructed from the genomic data.

## Organism information

### Classification and features

**Fig. 1:**
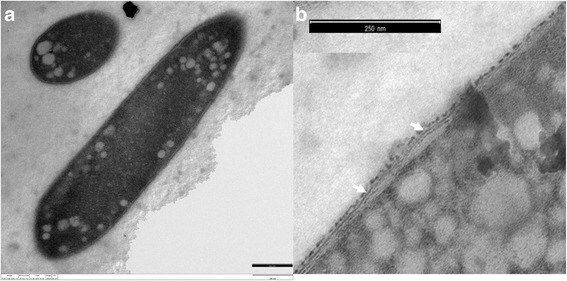
Transmission electron micrograph of *A. acetethylicum* strain GluBS11^T^ cells grown with gluconate. **a** Ultrathin trans-section of cell; **b** details of the Gram-positive membrane structure (*white arrows*)

*A. acetethylicum* strain GluBS11^T^ is a member of the family *Lachnospiraceae* in the phylum *Firmicutes* [19]. Cells were strictly anaerobic, non-motile and stained Gram-positive [1]. Fig. 1a shows the ultrathin trans-section of a rod-shaped cell and Fig. 1b shows details of the Gram-positive membrane structure. For transmission electron microscopy, fixation of bacterial cells was done with glutardialdehyde and osmium tetroxide followed by staining with uranylacetate. Samples were dehydrated in a graded ethanol series, embedded in Spurr resin and viewed in a Zeiss 912 Omega transmission electron microscope (Oberkochen, Germany) at 80 kV. Classification and general features are summarized in Table 1. Strain GluBS11^T^ ferments various substrates including glucose, lactose, sucrose, fructose, maltose, xylose, galactose, melibiose, melezitose, gluconate, mannitol, erythritol, glycerol and esculin, and mainly produces acetate, ethanol, hydrogen and formate as fermentation end products [1]. Although strain GluBS11^T^ was tested negative for catalase and peroxidase [1]. A gene coding for a putative catalase-peroxidase (IMG gene locus tag Ga0116910_10254) was identified in the draft genome. Besides this, strain GluBS11^T^ contains putative genes coding for thioredoxin reductase (Ga0116910_100846) and thioredoxin (Ga0116910_100229), and no gene coding for superoxide dismutase was identified in the genome. Strain GluBS11^T^ was tested positive for fermentation (API Rapid 32A reactions) of α-galactosidase, α-glucosidase and β-glucosidase [1]. The genome-based analysis identified genes coding for a putative β-galactosidase (Ga0116910_1001515 and Ga0116910_100295), a β-glucosidase (Ga0116910_100187 and Ga0116910_100196) and α-galactosidase (Ga0116910_10579, Ga0116910_100577 and Ga0116910_102538), respectively.

**Table 1:** Classification and general features of *Anaerobium acetethylicum* strain GluBS11^T^ according to the MIGS recommendations [53]

MIGS ID	Property	Term	Evidence code^a^
	Classification	Domain *Bacteria*	TAS [54]
		Phylum *Firmicutes*	TAS [19, 55]
		Class *Clostridia*	TAS [3, 56]
		Order *Clostridiales*	TAS [4, 57]
		Family *Lachnospiraceae*	TAS [2, 56]
		Genus *Anaerobium*	TAS [1]
		Species *Anaerobium acetethylicum*	TAS [1]
		Type strain: *GluBS11* ^*T*^ *(DSM 29698)*	
	Gram stain	positive	IDA, [1]
	Cell shape	rod-shaped	IDA, [1]
	Motility	non-motile	TAS [1]
	Sporulation	spore formation not reported	TAS [1]
	Temperature range	15-37 °C	IDA [1]
	Optimum temperature	30 °C	IDA, [1, 17]
	pH range; Optimum	3.5–6.5; 7.3	TAS [1]
	Carbon source	gluconate, glucose, glycerol	TAS [1, 17]
MIGS-6	Habitat	biogas slurry	TAS [1]
MIGS-6.3	Salinity	not determined	
MIGS-22	Oxygen requirement	anaerobe	TAS [1, 17]
MIGS-15	Biotic relationship	free-living	IDA
MIGS-14	Pathogenicity	non-pathogenic	NAS
MIGS-4	Geographic location	Germany	IDA
MIGS-5	Sample collection	2014	IDA
MIGS-4.1	Latitude	50.64 N	NAS
MIGS-4.2	Longitude	6.88 E	NAS
MIGS-4.4	Altitude	170 meter	NAS

^a^Evidence codes - IDA: Inferred from Direct Assay; TAS: Traceable Author Statement (i.e., a direct report exists in the literature); NAS: Non-traceable Author Statement (i.e., not directly observed for the living, isolated sample, but based on a generally accepted property for the species, or anecdotal evidence). These evidence codes are from the Gene Ontology project [58]

**Fig. 2:**
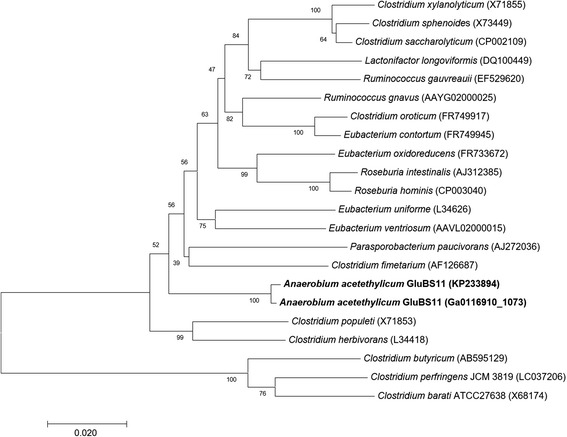
Phylogenetic tree constructed using MEGA 7 [20] showing the current position of the *A. acetethylicum* strain GluBS11^T^ with respect to the selected members from the order *Clostridiales*. The evolutionary distances were computed using the Jukes-Cantor method [22] and are in the units of the number of base substitutions per site. The phylogenetic tree was searched using the Close-Neighbor-Interchange algorithm [59] at a search level of 1. All positions containing gaps and missing data were eliminated. There were a total of 1,300 positions in the final dataset. Numbers at the nodes indicates the bootstrap values from 1000 replicates [60] and accession numbers are given in parentheses. Bar indicates 2% estimated sequence divergence

BLAST search results of the partial 16S rRNA gene sequence of *A. acetethylicum* strain GluBS11^T^ (KP233894) revealed closest sequence similarities with the uncultured *Lachnospiraceae* bacterium strain UY038 (94% similarity; HM099641) that was isolated from an oral sample, *C. populeti* ATCC 35295 ^T^ (94%; X71853) and *Robinsoniella* sp. MCWD5 (94%; KU886099). The draft genome sequence of *A. acetethylicum* GluBS11^T^ has one full-length 16S rRNA gene (1,536 bp; locus tag Ga0116910_1073) that was compared with the partial 16S rRNA gene sequence (1,402 bp; KP233894) from the original species description [1]. Sequence alignment indicated, that both 16S rRNA sequences were about 99% identical and the complete 16S rRNA gene sequence differs from the partial 16S rRNA gene sequence by the presence of an additional stretch of 45 bp long nucleotide sequence at the beginning, 5 gaps (53-55, 65 and 68 positions), and 9 base change at position 51 (T-A), 96 (G-A), 104 (A-T), 1,008 (T-A), 1,423 (A-T), 1,434 (A-G), 1,435 (T-G), 1,442 (A-C) and 1443 (T-C), followed by an additional long stretch of a 83 bp nucleotide sequence at the end. Figure 2 shows the current phylogenetic position of *A. acetethylicum* strain GluBS11^T^ in a phylogenetic tree constructed in MEGA 7 [20] using the Minimum Evolution method [21], and the evolutionary distances were computed using the Jukes-Cantor method [22] and the Neighbor-Joining algorithm [23].

## Genome sequencing information

### Genome project history

Strain GluBS11^T^ was selected for genome sequencing because of its ability to ferment gluconate or glycerol mainly to acetate, ethanol, hydrogen and small amounts of formate. Genome sequencing was performed through the community science program as part of the “Genomic Encyclopedia of Bacterial and Archaeal Type Strains, Phase III: the genomes of soil and plant-associated and newly described type strains” [24, 25]. The draft genome of *A. acetethylicum* strain GluBS11^T^ is listed in the Genomes OnLine Database under the GOLD project ID Gp0139288 [26], and the assembled and annotated high-quality permanent draft genome sequence is deposited in IMG under submission ID 88715 [27]. Whole genome shotgun sequencing project was also submitted to the Genbank/NCBI under the accession no., FMKA00000000 and consists of 105 contigs (FMKA01000001-FMKA01000105). Sequencing, finishing and annotation were performed by the Department of Energy, Joint Genome Institute using state-of-the-art sequencing technology [28]. A summary of the project information is shown in Table 2.

**Table 2:** Project information

MIGS ID	Property	Term
MIGS 31	Finishing quality	High-quality-draft
MIGS-28	Libraries used	An Illumina 300 bp insert standard shotgun (AZHBB)
MIGS 29	Sequencing platforms	Illumina HiSeq 2500-1 TB
MIGS 31.2	Fold coverage	336.0X
MIGS 30	Assemblers	SPAdes
MIGS 32	Gene calling method	Prodigal
	Locus Tag	BRJ36
	Genbank ID	FMKA00000000
	GenBank Date of Release	September 23, 2016
	GOLD ID	Gp0139288
	BioProject	PRJEB15475
MIGS 13	Source Material Identifier	GluBS11^T^ (= DSM 29698)
	Project relevance	Sugar and glycerol fermenting bacterium

### Growth conditions and genomic DNA preparation

*A. acetethylicum* strain GluBS11^T^ was cultivated in anoxic mineral medium supplemented with 10 mM gluconate as growth substrate at 30 °C for three days until OD_600nm_ 1.0 was reached. Genomic DNA was isolated from the cell pellet obtained from about 500 ml of grown culture using a CTAB-based method [29] with slight modifications [30]. After RNase treatment, the purity and quality of the genomic DNA preparation were assessed by DNA absorption at 260 nm and size by agarose gel electrophoresis (1% w/v; Additional file 1: Figure S1). The concentration of the isolated genomic DNA was 2.4 μg μl^-1^ (A_260/280_ = 2.03 and A_260/230_ = 2.47). Finally, the DNA was used to amplify the 16S rRNA gene to confirm the identity of genomic DNA by comparing with the described partial 16S rRNA gene sequence (KP233894) of *A. acetethylicum* strain GluBS11^T^. The pure and high-quality genomic DNA was shipped to the DOE, JGI for genome sequencing.

### Genome sequencing and assembly

The draft genome sequencing was performed at the DOE, JGI using the Illumina technology [31]. An Illumina 300 bp insert standard shotgun library was constructed and sequenced using the Illumina HiSeq-2500 1 TB platform, which generated 11,508,336 reads totaling 1,726.3 Mbp. All details on library construction and sequencing performed at the JGI can be found on the website. All raw Illumina sequence data were filtered using BBDuk [32], which removes known Illumina artifacts and PhiX. Reads with more than one “N” or with quality scores (before trimming) averaging less than 8 or reads shorter than 51 bp (after trimming) were discarded. Remaining reads were mapped to masked versions of human, cat and dog references using BBMap [32] and discarded if the identity exceeded 95%. Sequence masking was performed with BBMask [32]. The following steps were performed for assembly: (1) artifact filtered Illumina reads were assembled using the SPAdes genome assembler (version 3.6.2); [33], (2) assembly contigs were discarded if their length was <1 kbp. Parameters for the SPAdes assembly were -cov-cutoff auto -phred-offset 33 -t 8 -m 40 -careful -k 255,595 -12. The final draft assembly contained 108 contigs in 105 scaffolds, totaling 4.609 Mbp in size, and was based on 1,500.0 Mbp of Illumina data with a mapped coverage of 336.0X.

### Genome annotation

Genes were identified with Prodigal [34] using standard microbial genome annotation pipeline [35]. The predicted CDSs were translated and used to search the NCBI non-redundant database, UniProt, TIGRFam, Pfam, KEGG, COG, and InterPro databases. The tRNAScanSE tool [36] was used to find tRNA genes, whereas rRNA genes were found by searches against models of the rRNA genes built from SILVA [37]. Other non-coding RNAs such as the RNA components of the protein secretion complex and the RNase P were identified by searching the genome for the corresponding Rfam profiles using INFERNAL [38]. Additional gene prediction analysis and manual functional annotation (IMG taxon ID 2675903067) were performed within the Integrated Microbial Genomes-Expert Review platform [39] developed by the JGI, Walnut Creek, CA, USA.

## Genome properties

The draft genome sequence of *A. acetethylicum* strain GluBS11^T^ was based on an assembly of 105 DNA scaffolds (108 contigs) amounting to 4,609,043 (4.6 Mb) nucleotide base pairs with a calculated G + C content of 43.51 mol % (Table 3). Of the total of predicted CDSs of 4,132 genes (100%), 4,008 were assigned to protein-coding genes, of which 2,640 were assigned to COGs (63.89%), and the rest of 124 were assigned to RNA genes (3.0%). The majority of protein-coding genes (3,141 genes or 76.02%) were assigned to putative functions whilst the remaining genes were annotated as hypothetical proteins of unknown function. The draft genome properties, the statistics and the distribution of genes into COGs functional categories are summarized in Tables 3 and 4. The draft genome comparison of *A. acetethylicum* strain GluBS11^T^ using the BLASTn revealed top hits with the genomes of *C. nexile* DSM 1787 ^T^ (85% identity; NZ_DS995342.4), *Anaerostipes hadrus* DSM 3319 ^T^ (85%; NZ_KB290653.1), *Acetonema longum* DSM 6540 ^T^ (84%; NZ_AFGF01000168.1), *Anaerostipes caccae* DSM 14662 ^T^ (83%; NZ_DS499733.1), *Blautia hansenii* DSM 20583 ^T^ (83%; NZ_GG698589.1), and a ruman-associated strain, *Ruminococcus torques* ATCC 27756 ^T^ (82%; NZ_DS264349.1), and *C. phytofermentans* ATCC 700394 ^T^ (74%), respectively.

**Table 3:** Genome statistics

Attribute	Value	% of Total
Genome size (bp)	4,609,043	100
DNA coding (bp)	4,001,559	86.82
DNA G + C (bp)	2,005,619	43.51
DNA scaffolds	105	100
Total genes	4,132	100
Protein coding genes	4,008	97.00
RNA genes	124	3.00
Pseudo genes	867	20.98
Genes in internal clusters	1,252	30.30
Genes with function prediction	3,141	76.02
Genes assigned to COGs	2,633	63.72
Genes with Pfam domains	3,303	79.94
Genes with signal peptides	186	4.50
Genes with transmembrane helices	984	23.81
CRISPR repeats	0	0

The total is based on either the size of the genome in the base pairs or the total numbers of proteins coding genes in the annotated genome of *A. acetethylicum* GluBS11^T^

**Table 4:** Number of genes associated with general COG functional categories

Code	Value	%age	Description
J	199	6.69	Translation, ribosomal structure and biogenesis
A	-	-	RNA processing and modification
K	284	9.55	Transcription
L	121	4.07	Replication, recombination and repair
B	-	-	Chromatin structure and dynamics
D	32	1.08	Cell cycle control, Cell division, chromosome partitioning
V	57	1.92	Defense mechanisms
T	165	5.55	Signal transduction mechanisms
M	120	4.03	Cell wall/membrane biogenesis
N	63	2.12	Cell motility
U	43	1.45	Intracellular trafficking and secretion
O	89	2.99	Posttranslational modification, protein turnover, chaperones
C	174	5.85	Energy production and conversion
G	539	18.12	Carbohydrate transport and metabolism
E	224	7.53	Amino acid transport and metabolism
F	88	2.96	Nucleotide transport and metabolism
H	135	4.54	Coenzyme transport and metabolism
I	86	2.89	Lipid transport and metabolism
P	111	3.73	Inorganic ion transport and metabolism
Q	47	1.58	Secondary metabolites biosynthesis, transport and catabolism
R	233	7.83	General function prediction only
S	124	4.17	Function unknown
-	1,499	36.28	Not in COGs

The total is based on the total number of protein coding genes predicted in the genome of *A. acetethylicum* strain GluBS11^T^. – no data available

## Insights from the genome sequence

### General metabolic features

The draft genome of strain GluBS11^T^ was further examined to understand the organism’s physiology and fermentation metabolism. The draft genome encodes most of the key enzymes of the pentose phosphate pathway, Embden-Meyerhoff-Parnas pathway, Entner-Doudoroff pathway and tricarboxylic acid cycle (Additional file 2: Table S1). Thus, strain GluBS11^T^ is very likely to use these pathways for its central metabolism and biosynthesis. Besides this, the genome also contains the genes coding for putative enzymes of anaplerotic pathways, such as oxaloacetate decarboxylase (α-subunit, Ga0116910_1001318 and β-subunit, Ga0116910_1001319), pyruvate kinase (Ga0116910_1001611), fructose-1,6-bisphosphatase (Ga0116910_1001181 and 10346), phosphoenolpyruvate carboxykinase (Ga0116910_1001300) and pyruvate carboxylase β-subunit (Ga0116910_101716). Genes for biosynthesis of amino acids and most co-factors were also present (Additional file 2: Table S1).

Although cells of strain GluBS11^T^ are non-motile [1], the genome possesses genes that are predicted to encode flagellum components (Ga0116910_1001565, Ga0116910_1002133- Ga0116910_1002135, Ga0116910_100329, Ga0116910_1002133- Ga0116910_1002135) such as flagellar protein FliO/FliZ, flagellar motor switch protein FliN/FliY/FliM, flagellar FliL protein, and pilus assembly-protein (Flp/PilA), which are located in a single gene cluster (locus tag Ga0116910_100336 to Ga0116910_100363), including the chemotaxis protein (MotB/A). The draft genome also contains genes predicted to encode seven universal stress proteins of the UspA family (gene loci Ga0116910_103114, 1003225, 10025, 10028, 10027, 104111 and 100540), 2 heat-shock proteins such as GrpE (Ga0116910_10476 and 100386), one heat-inducible transcriptional repressor (Ga0116910_100387), and six cold-shock proteins of the CspA family (Ga0116910_10067, 1002200, 1001175, 1004187, 1005160 and 1002190). Also, a DNA-directed RNA polymerase with sigma-70/32 factor (ECF family) and a heat-inducible transcriptional repressor (HrcA) along with the RNA polymerase sigma factor for flagellar operon FliA were detected in the draft genome.

Clustered regularly interspaced short palindromic repeats are segments of prokaryotic DNA containing short repetitions of base sequences followed by a short segment of ‘spacer DNA’ that function as a defense system against the introduction of foreign genetic materials (e.g., phage infection, plasmid or horizontal gene transfer). CRISPRs were found in approximately 40% of all sequenced bacterial genomes [40]. Genome analysis of strain GluBS11^T^ suggests that the genome does not contain CRISPR regions, although the genome of the phylogenetically closely related strain *C. populeti* ATCC 35295 ^T^ contains two gene coding for CRISPR-associated proteins (cas9 family protein; Ga0056054_02523 and Ga0056054_00025).

### Transporters

Transporters enable bacteria to accumulate required nutrients and also contribute for excretion of unwanted metabolic products. They also help to maintain the osmotic balance and the cytoplasmic pH by transporting H^+^ and various salts. Genome analysis of strain GluBS11^T^ identified various membrane transporters including the ABC solute transporters (ATP-dependent) that could take part in the transport of various substrates such as ions, vitamins, sugars, amino acids, and metabolites (Additional file 3: Table S2). Most of these identified transporters belong to diverse transporter families such as the amino acid/polyamine antiporter family, the drug/metabolite transporter superfamily, and the major facilitator superfamily that is used for transport of a diverse set of small solutes in response to chemiosmotic ion gradients [41]. The draft genome sequence also contains several genes coding for proton symporters (Additional file 3: Table S2). Thus, strain GluBS11^T^ could generate a proton gradient using FoF_1_-type ATP synthase in reverse direction [42, 43].

### Metabolic pathways for glucose, gluconate and glycerol utilization

**Fig. 3:**
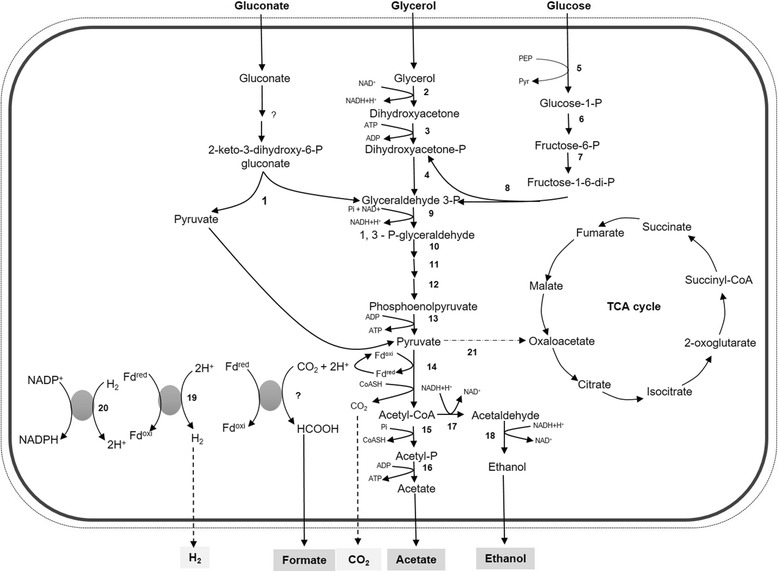
Metabolic network of glucose and gluconate, including glycerol [17] metabolism by *A. acetethylicum* strain GluBS11^T^ reconstructed from the IMG annotated draft genome sequence. Numbers adjacent to arrows represent putative enzymes. 1) 2-keto-3-deoxphosphogluconate aldolase (locus tag, Ga0116910_101517); 2) glycerol dehydrogenase (Ga0116910_101526 and 101551); 3) dihydroxyacetone kinase (Ga0116910_ 1001186, 1001188, 101527, 101552 and 101085); 4) triosephosphate isomerase (Ga0116910_ 1001390, 102914, 101435 and 101134); 5) phosphotransferase system (PTS; Ga0116910_100991 and Ga0116910_100370); 6) phosphogluconomutase (Ga0116910_ 1007105, 10644, 1002181 and 10031112); 7) phosphofructokinase (Ga0116910_100239); 8) fructose 1, 6-bisphosphate aldolase (Ga0116910_100167); 9) glyceraldehyde 3-phosphate dehydrogenase (Ga0116910_1001391); 10) phosphoglycerate kinase (Ga0116910_1001391); 11) phosphoglycerate mutase (Ga0116910_1001389 and Ga0116910_103027); 12) enolase (Ga0116910_1001503); 13) pyruvate kinase (Ga0116910_1004153); 14) pyruvate ferredoxin oxidoreductase (Ga0116910_103224 and Ga0116910_101718); 15) phosphoacetyl transferase (Ga0116910_1001587); 16) acetate kinase (Ga0116910_1001586); 17) CoA-dependent acetaldehyde dehydrogenase (Ga0116910_1004188); 18) alcohol dehydrogenase (Ga0116910_101528 and 101313); 19) iron-only hydrogenases (Ga0116910_100545, Ga0116910_1001473 and Ga0116910_100543); 20) NADP-reducing hydrogenases (Ga0116910_1001466,Ga0116910_1001467, Ga0116910_1001468, Ga0116910_1001470) and 21) putative pyruvate carboxylase (Ga0116910_101716)

Strain GluBS11^T^ ferments sugars, e.g., glucose and gluconate or glycerol mainly to ethanol and hydrogen, including the production of acetate and small amounts of formate as fermentation end products [1, 17]. In the present study, a metabolic network for the utilization of glucose and gluconate including glycerol was constructed based on the genome as shown in Fig. 3, from the genome annotation provided by the IMG-ER. To determine which pathway was utilized for glycerol fermentation, a recent study by Patil et al., [17] provided insight into glycerol fermentation of strain GluBS11^T^ using biochemical and proteomic approaches. There are three possible alternatives for gluconate metabolism: first, the phosphorylation to gluconate 6-phosphate (the Entner-Doudoroff pathway), second, the reduction to glucose or lastly, the dehydration to 2-keto-3-deoxy-gluconate, a modified Entner-Doudoroff pathway [44]. In the last four decades, several studies reported that gluconate fermentation by numerous anaerobic bacteria, e.g., *Clostridium aceticum* DSM 1496 ^T^ [45] or *E. coli* ML30 (DSM 1328 ^T^); [46] proceeds through a modified Entner-Doudoroff pathway.

The genome annotation predicted the presence of four gluconate:proton symporters (Gnt family) encoded by Ga0116910_10413, Ga0116910_10069, Ga0116910_100214 and Ga0116910_10418. In a previous study, it was shown that *C. acetobutylicum* ATCC 824 ^T^ takes up gluconate by gluconate:proton symporters (CA_C2835); [47] which showed amino acid sequence identity (24 to 41%) with the four predicted genes with highest identity (Ga0116910_10418; 42%). Thus, the product of the Ga0116910_10418 gene is the most likely candidate for uptake of gluconate. Based on the genome annotation, *A. acetethylicum* strain GluBS11^T^ most likely uses the Entner-Doudoroff pathway for gluconate metabolism, through which gluconate is first phosphorylated to 6-phosphogluconate by gluconokinase (EC 2.7.1.12) followed by dehydration to 2-keto-3-deoxy-phosphogluconate by 6-phosphogluconate dehydratase (EC 4.2.1.12). Alternatively, gluconate could be first dehydrated (modified Entner-Doudoroff pathway) to 2-keto-3-deoxy gluconate by gluconate dehydratase (EC 4.2.1.39) followed by phosphorylation to KDPG by 2-keto-3-deoxygluconokinase (EC 2.7.1.45). KDPG would be further converted to pyruvate and glyceraldehyde 3-phosphate by KDPG aldolase (EC 4.1.2.14). The presence of a putative gene coding for KDPG aldolase (Ga0116910_101517) indicates that gluconate is most likely metabolized via KDPG. However, no putative genes coding for the initial enzymes that could convert gluconate to KDPG (according to two ways as mentioned above) was identified in the draft genome of strain GluBS11^T^. However, two putative genes were annotated as dihydroxy acid dehydratase/phosphogluconate dehydratase (Ga0116910_10068 and Ga0116910_101679) that could have this activity. The predicted dihydroxy acid dehydratase (EC 4.2.1.9) is possibly involved in the biosynthesis of amino acids (valine, isoleucine, and isoleucine). A similar observation was also reported for the gluconate-fermenting *C. acetobutylicum* ATCC 824 ^T^, where the gene CA_C3170 was predicted to encode a 6-phosphogluconate dehydratase and BlastP analysis indicated that it is a dihydroxy acid dehydratase primarily involved in the synthesis of amino acids [47, 48]. BlastP search of amino acid sequence analysis of genes Ga0116910_10068 and Ga0116910_101679 showed more than 80% identity with the dihydroxy acid dehydratase of *C. phytofermentans* ATCC 700394 ^T^ (A9KL28) and *Anaerostipes caccae* DSM 14662 ^T^, respectively, and showed only 40-60% identity with gene CA_C3170 of *C. acetobutylicum* ATCC 824 ^T^. Therefore, genes Ga0116910_10068 and Ga0116910_101679 most likely encode a dihydroxy acid dehydratase that is involved in amino acid synthesis rather than in KDPG formation. Based on this information, gluconate degradation via the Entner-Doudoroff pathway involving gluconate phosphorylation to 6-phosphogluconate by gluconokinase (EC 2.7.1.12) followed by dehydration to KDPG by 6-phosphogluconate dehydratase (EC 4.2.1.12) can be ruled out. Furthermore, the presence of a putative gene coding for KDPG aldolase (Ga0116910_101517) indicates that gluconate is most likely metabolized via the modified Entner-Doudoroff pathway, which would be consistent with previous studies of the anaerobic gluconate metabolism [45, 47, 49]. Even though no genes coding for the gluconate dehydratase (EC 4.2.1.39) and KDG kinase (EC 2.7.1.178) required for initial activation of gluconate to KDPG were identified in the genome of strain GluBS11^T^.

While gluconate is predicted to be metabolized via the modified Entner-Doudoroff pathway, glucose could be metabolized through glycolysis. For uptake of glucose, strain GluBS11^T^ most likely uses a phosphotransferase system (PTS) which couples glucose import to its phosphorylation with phosphoenolpyruvate, yielding glucose-6-phosphate and pyruvate [47]. Genes Ga0116910_100991 and Ga0116910_100370 are predicted to encode PTS proteins which are most likely involved in glucose transport in strain GluBS11^T^. Thus, genome analysis suggests that glucose is most probably metabolized through glycolysis via glucose 6-phosphate by glucose-6-phosphate isomerase (Ga0116910_1004120 and Ga0116910_10539), 6-phosphofructokinase (Ga0116910_103531, Ga0116910_100239, Ga0116910_102039 and Ga0116910_101135), and fructose-bisphosphate aldolase (Ga0116910_101128 and Ga0116910_102024) to glyceraldehyde 3-phosphate. In the glycolysis pathway, glyceraldehyde 3-phosphate is further metabolized through the lower part of glycolysis to ethanol, acetate, hydrogen, and formate. During gluconate fermentation, KDPG aldolase would then convert KDPG to glyceraldehyde-3-phosphate and pyruvate, and only glyceraldehyde-3-phosphate passes through the lower glycolysis pathway.

Previous studies with other bacteria reported that gluconate fermentation mainly yielded acetate and butyrate as fermentation products [45, 47, 49]. Although, the draft genome of strain GluBS11^T^ contains genes predicted to code for a putative butyrate kinase (Ga0116910_101723 and Ga0116910_102110), gluconate, glucose or glycerol fermentation by strain GluBS11^T^ does not produce butyrate [1, 17]. The pathways were easily constructed based on the genome analysis and genes for acetate metabolism, e.g., acetate kinase (Ga0116910_103636, Ga0116910_1001586 and Ga0116910_104214), ethanol metabolism, e.g., alcohol hydrogenase (Ga0116910_101528, Ga0116910_102038, Ga0116910_102215, Ga0116910_1004154 and Ga0116910_102016), and hydrogen metabolism, e.g., putative iron-only hydrogenases and subunits coding for an NADP^+^-reducing hydrogenase (Ga0116910_1001473, Ga0116910_1001466, Ga0116910_1001467, Ga0116910_1001468, Ga0116910_1001470, Ga0116910_100545 and Ga0116910_1001473). No candidate gene was found to code for a putative formate-producing formate dehydrogenase in the draft genome of strain GluBS11^T^ even though formate dehydrogenase activities were detected in cell-free extracts using benzyl viologen as an artificial electron acceptor [17]. On the other hand, genes annotated as pyruvate:formate lyase or formate C-acetyltransferase were identified in the genome (Ga0116910_1004109, Ga0116910_100860, Ga0116910_102934 and Ga0116910_102935), but no activity for a possible pyruvate:formate lyase could be detected [Patil et al., unpublished results]. This indicates that the genomic information is sometimes insufficient to predict metabolic pathways. Thus, further biochemical and proteomics studies would be needed to investigate and confirm the gluconate and glucose fermentation pathway utilized by this bacterium.

### Microcompartments and fucose utilization

The genome of *A. acetethylicum* strain GluBS11^T^ harbors five genes that putatively code for bacterial microcompartment shell proteins. Four of these genes are annotated as “BMC-domain-containing protein” (Ga0116910_1005148, Ga0116910_1005149, Ga0116910_1005150 and Ga0116910_1005151), and one gene is annotated as “Carboxysome shell and ethanolamine utilization microcompartment protein CcmL/EutN” (Ga0116910_1005155). Microcompartments are protein complexes that form discrete spaces within the cell, thus enabling enzyme reactions that either produce toxic intermediates or require accumulation of a certain metabolite, e.g., the ethanolamine utilization microcompartment in *Salmonella typhimurium* ATCC 13311 ^T^ or the carboxysomes in cyanobacteria [50, 51]. An IMG gene search for microcompartments and subsequent comparison to other genomes using the IMG Gene Ortholog Neighborhoods viewer, revealed that the microcompartment genes in *A. acetethylicum* strain GluBS11^T^ are located in a putative operon that also contains genes associated with fucose utilization in *Clostridium phytofermentans* ATCC 700394 ^T^ [52]. Fucose, a deoxyhexose derived from plant biomass degradation, can be fermented to propionate, propanol, mixed acids, and ethanol by *C. phytofermentans* ATCC 700394 ^T^ *,* and the responsible genes are located in two different operons in this organism [52]. Initially, fucose is converted to fuculose-phosphate by fucose mutarotase, fucose isomerase and fucose kinase (Cphy_3153 – Cphy_3155); [52]. Likewise, the orthologs in *A. acetethylicum* strain GluBS11^T^ are located in a similar operon (L-fucose isomerase Ga0116910_100812, rhamnulokinase/L-fuculokinase Ga0116910_100813 and L-fucose mutarotase Ga0116910_100815). Fuculose-phosphate is then further degraded to lactaldehyde and dihydroxyacetone-phosphate by fuculose-phosphate aldolase (Ga0116910_102223 in *A. acetethylicum* strain GluBS11^T^, Cphy_1177 in *C. phytofermentans*). Dihydroxyacetone phosphate can then be processed through glycolysis, while lactaldehyde is reduced to 1,2-propanediol with NADH. 1,2-propanediol is then disproportionated in microcompartments to propionate and propanol by 1,2-propanediol oxidoreductase (Cphy_1185, Ga0116910_1005154), 1,2-propanediol dehydratase (Cphy_1174, Ga0116910_100557 - Ga0116910_100559 in a different area of the genome), propionaldehyde dehydrogenase (Cphy_1178, Ga0116910_1005146), phosphate propanoyl transferase (Cphy_1183, Ga0116910_1005152), acetate/propionate kinase (Cphy_1327, Ga0116910_104214, Ga0116910_1001586, or Ga0116910_103636) and propanol dehydrogenase (Cphy_1179, Ga0116910_1005147). Rhamnose can be degraded in a similar way by *C. phytofermentans* ATCC 700394 ^T^ *,* and the respective genes leading to lactaldehyde and dihydroxyacetone-phosphate were also identified in the genome of *A. acetethylicum* strain GluBS11^T^ (L-rhamnose mutarotase Ga0116910_10513, L-rhamnose isomerase Ga0116910_1001301, rhamnulokinase/L-fuculokinase Ga0116910_100813) [52]. However, earlier results demonstrated that rhamnose cannot be utilized by *A. acetehylicum* strain GluBS11^T^ [52]. Even though the genes for fucose degradation are present in the genome, it is still doubtful whether this sugar can serve as a growth-supporting substrate for strain GluBS11^T^.

## Conclusions

Taken together, the draft genome sequence of *A. acetethylicum* strain GluBS11^T^ expands our view on the metabolic capacities of this sugars and glycerol-fermenting bacterium. The genome sequence gives us insights into the putative enzymes involved in the pathway of glucose and gluconate (including glycerol) fermentation, and provides a brief summary of the key reactions involved. Lastly, the hypotheses concerning the glucose and gluconate fermentation pathways based on genomic data are still preliminary, and additional biochemical and functional proteomic studies will be necessary for pathway confirmation and further insights.

## Additional files

Additional file 1: Figure S1. Gel electrophoresis of genomic DNA isolated from GluBS11^T^ cells grown with gluconate. (TIF 159 kb)
Additional file 2: Table S1. IMG annotated functions of selected putative key enzymes involved in the metabolic pathways identified in the draft genome sequence of *A. acetethylicum* strain GluBS11^T^. (DOCX 15 kb)
Additional file 3: Table S2. Putative transporters identified in the draft genome of *A. acetethylicum* GluBS11^T^. (DOCX 14 kb)

## Abbreviations

CDS: Coding DNA sequence
COG: Clusters of orthologous groups
CTAB: Cetyl trimethyl ammonium bromide
KEGG: Kyoto encyclopedia of genes and genomes
MEGA: Molecular evolutionary genetics analysis
NADH: Nicotinamide adenine dinucleotide reduced
