# Supplementary figures and images for: High-quality-draft genome sequence of the fermenting bacterium Anaerobium acetethylicum type strain GluBS11T (DSM 29698)
Source: Stand Genomic Sci. 2017 Feb 20;12:24. doi: 10.1186/s40793-017-0236-4 (PMC5322786; doi:10.1186/s40793-017-0236-4)

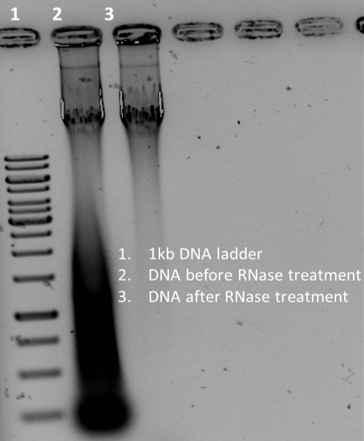

Supplement: Additional file 1: Figure S1. — Gel electrophoresis of genomic DNA isolated from GluBS11T cells grown with gluconate. (TIF 159 kb) [file 40793_2017_236_MOESM1_ESM.tif]
