# Supplementary material for: High-quality-draft genome sequence of the fermenting bacterium Anaerobium acetethylicum type strain GluBS11T (DSM 29698)
Source: Stand Genomic Sci. 2017 Feb 20;12:24. doi: 10.1186/s40793-017-0236-4 (PMC5322786; doi:10.1186/s40793-017-0236-4)
Supplement: Additional file 2: Table S1. — IMG annotated functions of selected putative key enzymes involved in the metabolic pathways identified in the draft genome sequence of A. acetethylicum strain GluBS11T. (DOCX 15 kb) [file 40793_2017_236_MOESM2_ESM.docx]

**Table S1.** IMG annoatated functions of selected putative key enzymes involved in the metabolic pathways identified in the draft genome sequence of *A. acetethylicum* strain GluBS11^T^.

| **Main pathways** | **Key enzymes identified** | **Gene loci**  (Ga0116910_ **)** |
| --- | --- | --- |
| Glycolysis | Phosphofructokinase  Fructose 1, 6-biphosphate aldolase  Pyruvate kinase | 100239  101258  100167 |
| Pentose phosphate pathway | Gluconolactonase  Ribulose-5-phosphate 3-epimerase  Transketolase  Transaldolase | 1001376  100910, 10099, 103543  10533, 103542  101534 |
| ED pathway | 2-keto-3-deoxphosphogluconate aldolase | 101517 |
| TCA cycle | Malate dehydrogenase  Citrate synthase  Aconitase  Fumarate hydratase αβ subunit  Isocitrate dehydrogenase (NAD+)  Pyruvate-ferredoxin/flavodoxin oxidoreductase Oxaloacetate decarboxylase, alpha subunit  Pyruvate carboxylase subunit B | 103828  1001297  1004146  1004208, 100771, 100772, 1004209  1001415  1005157, 103224, 101718  1001318  101716 |
| Anaplerotic pathway | Pyruvate carboxylase subunit B  Oxaloacetate decarboxylase, alpha subunit  Malate dehydrogenase (malic enzyme) | 101716  1001318  1002215 |
| Amino acid metabolism | Tryptophan synthase, alpha chain  Tryptophan synthase beta chain/phosphoribosyl anthranilate isomerase  Anthranilate phosphoribosyl transferase  Phosphoribosyl anthranilate isomerase  Histidinol dehydrogenase  Histidyl-tRNA synthetase  Glutamine amido transferase  Dihydroxy-acid dehydratase  Threonine synthase  Threonine dehydratase  L-threonine aldolase | 1005144  1005143  10594  1005142  1001211  1008100  1001617  10068  1001330  1001578  10256 |
| Lipid metabolism | Glycerol kinase  glycerol-3-phosphate acyltransferase PlsX, PlsY  Diacylglycerol kinase (ATP) | 1005138, 104021  1001583, 1001559  1001177, 101168 |
| Glycerol metabolism | Glycerol dehydrogenase  Dihydroxyacetone kinase | 101526, 101551  101527 |
| Acetate metabolism | Phosphoacetyl transferase  Acetate kinase | 1001587  1001586 |
| Ethanol metabolism | Alcohol dehydrogenase  Phosphoacetyl transferase | 101528, 101313  1001587 |
