# Supplementary material for: High-quality-draft genome sequence of the fermenting bacterium Anaerobium acetethylicum type strain GluBS11T (DSM 29698)
Source: Stand Genomic Sci. 2017 Feb 20;12:24. doi: 10.1186/s40793-017-0236-4 (PMC5322786; doi:10.1186/s40793-017-0236-4)
Supplement: Additional file 3: Table S2. — Putative transporters identified in the draft genome of A. acetethylicum GluBS11T. (DOCX 14 kb) [file 40793_2017_236_MOESM3_ESM.docx]

**Table S2.** Putative transporters identified in the draft genome of *A. acetethylicum* GluBS11^T^.

| **Locus tag**  Ga0116910_ | **IMG annotated putative function** |
| --- | --- |
| 100948 and 1003216  100946 and 1003223  100947 and 1003224  100949  100941 and 1003220  100944  1003222  100945  1003221 | F-type H+-transporting ATPase β subunit  F-type H+-transporting ATPase α subunit  F-type H+-transporting ATPase γ subunit  F-type H+-transporting ATPase ε subunit  F-type H+-transporting ATPase α subunit  F-type H+-transporting ATPase β subunit  ATP synthase, F0 β subunit  F-type H+-transporting ATPase β subunit  F-type H+-transporting ATPase c subunit |
| 1001457  101710  1001459, 1001490, 101535, 101643 | basic amino acid/polyamine antiporter, APA family  Threonine/homoserine efflux transporter RhtA  Permease of the drug/metabolite transporter (DMT) superfamily |
| 1001465, 101045 1001610 | Formate/nitrite transporter FocA, FNT family  Succinate-acetate transporter protein |
| 101480  101478, 101479  1004161 | Energy-coupling factor transport system permease protein  Energy-coupling factor transporter ATP-binding protein EcfA2  Energy-coupling factor transporter transmembrane protein EcfT |
| 101945, 10248,  101156  1001613, 101280 1004168  1001353  101328  100689  1001269  101142, 101141, 1001269 | zinc transporter, ZIP family/zinc and cadmium transporter  cation diffusion facilitator family transporter  magnesium transporter  metal ion transporters  MFS transporter, UMF1 family  MFS-type transporter involved in bile tolerance, Atg22 family  MFS transporter, DHA3 family, macrolide efflux protein  sulfate permease, SulP family |
| 10648, 100776, 103213 102731, 102841  10279, 101232, 1003206, 100796  100186, 10275 | Na+/melibiose symporter or related transporter  Glycoside/pentoside/hexuronide:cation symporter, GPH family/probable glucitol transport protein GutA  Probable glucitol transport protein GutA |
| 1001150, 1005134  10308  100156, 101276  100698  1001413 | ATPase components of ABC transporters with duplicated ATPase domains  ATP-binding cassette, subfamily F, member 3  Di- and tricarboxylate transporter  lincosamide and streptogramin A transport system ATP-binding/permease protein  ATP-binding cassette, subfamily F, member 3 |
| 102822  102820 | putative tricarboxylic transport membrane protein  Tripartite-type tricarboxylate transporter, receptor component TctC |
|  |  |
|  |  |
|  |  |
|  |  |
| 1005156, 100912 | Biotin transport system substrate-specific component |
